# Introducing the ‘3 Fs model of complexity’ for people with dementia accessing a NHS mental health inpatient dementia assessment ward: An interpretive description study

**DOI:** 10.1177/14713012221136313

**Published:** 2022-11-08

**Authors:** Lesley Jones, Nicky Cullum, Ruth Watson, John Keady

**Affiliations:** Head of Mental Health, Learning Disability and Autism, 53244 North West Ambulance Service NHS Trust, Manchester, UK; Professor of Nursing, Division of Nursing, Midwifery and Social Work, 5292 The University of Manchester, Manchester, UK; Principal Clinical Psychologist, 9022 Greater Manchester Mental Health NHS Foundation Trust, Manchester, UK; Division of Nursing, Midwifery and Social Work/Greater Manchester Mental Health NHS Foundation Trust, 5292 The University of Manchester, Manchester, UK

**Keywords:** complexity, inpatient assessment, dementia, practice model, interpretive description

## Abstract

**Background:**

In the United Kingdom, the use of the terms ‘complex’ and ‘complexity’ alongside dementia is reflected in a number of policy and practice documents. However, there is a lack of evidence that explores how complexity is perceived, constructed and experienced by people with dementia, family carers and practitioners working in the NHS dementia inpatient assessment wards [dementia assessment wards].

**Objective:**

To explore the meaning and concept of complexity in dementia from within the setting of a dementia assessment ward and develop a practice model.

**Methods:**

The study was conducted over three phases: 1) an online electronic survey of UK national dementia leaders; 2) individual interviews and a focus group with dementia practitioners in two dementia assessment wards; 3) case studies of four patients with dementia resident on a dementia assessment ward which included their identified family carer/consultee, the named clinician on the ward involved in that person’s care and a care records review.

**Results:**

The findings highlighted that complexity is constructed through a number of interconnected and interrelated domains that vary in acuity. These findings have been developed into the ‘3 Fs Model of Complexity’ and the 3‘Fs’ stand for Fixed, Flexible and Fluctuating. The Fixed domain consists of six components which are always present in complexity. The Flexible domain consists of 14 components and a person with dementia may experience any number of Flexible domain components at any one time. The Fluctuating domain highlights that all components have the ability to vary in their acuity.

**Conclusion:**

The ‘3 Fs Model of Complexity’ may facilitate a more holistic view of a person with dementia than when ‘symptoms’ are viewed in isolation. Going forward, and subject to further refinement and testing, the ‘3 Fs Model of Complexity’ could help guide the selection of tailored, personalised interventions for people with dementia, including formulation approaches.

## Introduction

In the United Kingdom (UK) a National Health Service (NHS) mental health inpatient dementia assessment ward (‘dementia assessment ward’ hereafter) is usually part of a specialist hospital service provided by NHS Mental Health Trusts for older people. For people with dementia, their admission to a dementia assessment ward is almost invariably made compulsorily under a section of either the Mental Health Act (Department of Health, 1983) or the requirements of the Mental Capacity Act (Department of Health, 2005) owing to issues around the person’s informed consent, capacity and assessed levels of risk in the community. The dementia assessment ward is an environment where ‘old age’ psychiatry and mental health professionals directly manage care provision and where it is the impact of the person’s dementia on their actions and behaviours that is the main clinical reason for admission (Royal College of Psychiatrists, 2006). Dementia assessment wards are not to be confused with specialist inpatient dementia assessment units (SIDUs) that are purposively set up in NHS acute hospitals to treat people with dementia admitted for a range of physical conditions, such as a fractured neck of femur, with that physical healthcare need being the main clinical reason for admission (for a review see: McCausland et al., 2019). The uncertainty over the operational functioning of a dementia assessment ward is perhaps understandable as both national strategy documents that underpinned the Prime Minister’s Challenge on Dementia 2020 (Department of Health, 2012, 2015), and its most recent implementation plan (Department of Health, 2016), failed to mention needs, or care provision, on a dementia assessment ward, concentrating instead on the acute hospital setting.

Admission to a dementia assessment ward is usually triggered by the person with dementia’s changed behaviour at home or in a supported living environment in the community, such as a care home, coupled with an inability to safely ‘manage’ the associated risk(s) in that existing location of care. For the person with dementia, such changed behaviour(s) could include the onset, or prolonged experience, of agitation, intrusiveness, restlessness, continence problems, eating difficulties and excessive vocalisation, for instance (Gray et al., 2022). This is by no means a complete list of changed behaviours and it is known that around 90% of all people with dementia will experience one, if not more, of these changed behaviours during the course of their dementia, especially during its later stages (Pinner et al., 2011). However, what is less predictable for the person with dementia is the duration and acuity of such changed behaviour(s) and the resultant impact on personal safety and risk (James & Jackman, 2017). Furthermore, family carer exhaustion, or the collapse of the support system at home that surrounds the person with dementia, has also been identified as a potential trigger point that could lead a person with dementia to be compulsorily admitted from home to a dementia assessment ward (Ross & Dexter-Smith, 2017).

Given the vulnerability of the person with dementia in such circumstances, it would be natural to assume that these environments of care have been subjected to a number of in-depth and detailed programmes of work. However, this does not appear to be the case and the literature involving dementia assessment ward practice reveals a wide-range of diverse and disparate studies that stretch from the use of life story work in improving care (Gridley et al., 2016), establishing the feasibility of using virtual reality within the ward setting (Rose et al., 2021), medication audits (Wilson et al., 2015), duration of stay (Ball et al., 2004), outcomes of admission (Wattis et al., 1994), physical morbidity (Adamis & Ball, 2000), formulation approaches (Keady & Jones, 2010), psychological consultation (Murphy et al., 2013) and representation via theatre production (Schneider, 2017). Moreover, the dementia assessment ward has also been used as the location of a sociological study on hair and care for people with dementia (Ward et al., 2016). Where there is a more cohesive thread in the literature is in the narrative that people with dementia pre- and post-admission to a dementia assessment ward are seen to be ‘complex’ in their presentation (Ross & Dexter-Smith, 2017; Royal College of Psychiatrists, 2006, 2015). However, what constitutes complexity in such circumstances is unclear as is how complexity transfers and translates into routine everyday practice on a dementia assessment ward.

Developing these points further, in England, the first National Institute for Health and Clinical Excellence/Social Care Institute for Excellence (2006) dementia guideline referred to complexity in dementia as being associated with the ‘coexistence of physical and psychiatric problems’ (p.34), which the guideline suggested may require assessment in a dementia assessment ward. Interestingly, the most recent National Institute for Health and Care Excellence (NICE) dementia guideline (NICE, 2018), which updated and replaced the 2006 one, omitted this reference to complexity and potential dementia assessment ward admission and, instead, briefly referred to the provision of care and support as being ‘complex’. This latter positioning was ascribed to the prevalence of dementia in the UK, which is currently estimated to be around 940,000 people (Wittenberg et al., 2019), and aligned it to the variation(s) in diagnosis, signs and symptoms that each person might experience. In contrast, studies by Sonola et al. (2013) and McGeorge (2010) have specifically looked at complexity in dementia and highlighted its dynamic, fluid and interactional properties and its relationship with physical, psychological, functional, environmental and social factors. McGeorge’s (2010) study in particular suggested that a state of complexity for people with dementia had the potential for a degree of recovery, but that this was complicated by the unpredictability, instability and intangibility of certain ‘internal’ factors experienced by a person with dementia, such as the severity and duration of their changed behaviour(s) and its relationship to any underlying physical health needs. McGeorge (2010), whose work was conducted in a single NHS Mental Health Trust in the UK with 13 members of staff, including those on a dementia assessment ward, also found that complexity was difficult to assess, measure and discuss owing to the lack of existing vocabulary and agreed terminology to assist practice.

In an attempt to further develop McGeorge’s (2010) insightful contribution to the literature, the primary aim of this interpretive description study (Thorne, 2008) was to explore the meaning and concept of complexity in dementia from within the setting of a dementia assessment ward.

## Design and Methods

### Design

The primary aim of the study was supported by four objectives:

 (i) To explore with various stakeholders in dementia care, including clinical staff, how they construct and recognise complexity in dementia.
 (ii) To explore the perspectives and views of complexity by people with dementia together with their main care support at home.
 (iii) To describe the key components of complexity in dementia.
 (iv) To build a practice model of complexity for future empirical testing and refinement.

**Figure 1.**
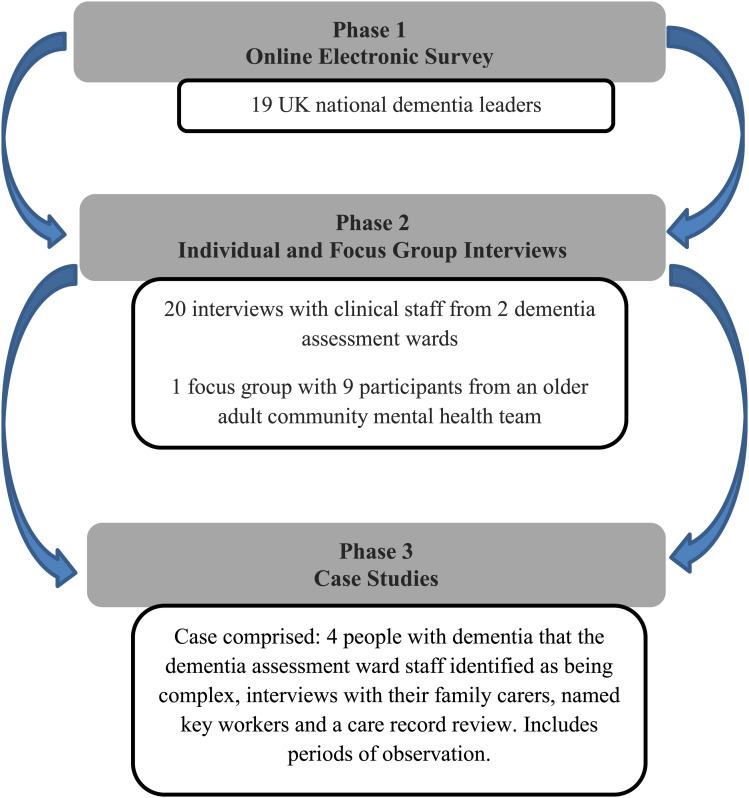
Summary of the study phases and methods.

To meet the study aim and objectives, a three-phase, mixed methods study design was implemented (see Figure 1) and data were collected and analysed between June 2017 to December 2018. The methods included electronic survey, in-person semi-structured interviews, observations, documentary analysis of participant (people with dementia) care records and researcher reflections. The latter aspect was supported by the lead author/researcher being a highly specialist mental health nurse who had worked on dementia assessment wards for over 20 years.

As seen in Figure 1, each phase of the study was iterative so that knowledge generated from phase 1 informed the approach to phase 2 and so on. In phases 2 and 3, participants were recruited from two dementia assessment wards in one NHS Mental Health Trust in the north of England. In line with the study protocol, these two wards have been ascribed pseudonyms which we have chosen as ‘Daisy Ward’ and ‘Jasmine Ward’. Daisy Ward and Jasmine Wards were in different geographic locations in the participating NHS Mental Health Trust and admitted people with dementia from different parts of the county.

Ethical approval to conduct this study was obtained from the North West Haydock NHS Research Ethics Committee (reference: 15/NW/0116). All data are anonymised in this article in line with the study protocol and all participants consented to take part. In phase 3 of the study, where concerns were expressed with regards to an individual’s capacity to consent, capacity assessments were completed in line with the study protocol. The consent process was carried out following the Mental Capacity Act (Department of Health, 2005) and inviting a relevant consultee to consider providing consent on behalf of the identified participant.

### Data collection activities and participants

Phase 1 of data collection activities comprised the distribution a two-stage e-survey. Questions for the e-survey were formed by all authors of this article with input from the study advisory group. Stage 1 of the e-survey comprised of background and demographic information from participants and stage 2 asked for free-text answers to the following four questions:

 (i) In your experience, what are the reasons people with dementia are admitted to a dementia assessment ward?
 (ii) What is your understanding of the term complexity in the context of dementia?
 (iii) What factors do you feel contribute to complexity in dementia?
 (iv) How should people with a complex presentation of dementia be assessed in a dementia assessment ward?

The e-survey was developed so that it would be easy to access on ‘SelectSurvey’ and take no longer than 30 min to complete. A decision was made to limit participant recruitment to those who worked in the UK as those outside of the UK may not be able to identify with the operational practices of a dementia assessment ward. The inclusion criteria centred on individuals who were leading clinicians, policymakers, academics and people with lived experience with participants having a publicly available email address and an ability to meet at least one of the following criteria:

• An academic in dementia care/studies (university based role).
• A lead dementia role in a care organisation (NHS or care homes).
• A dementia advisory position that influences, or has influenced, UK national policy and guidance.
• Be widely published on the topic of dementia care; and/or
• Be a person with dementia who does public-facing work on dementia and who has experience of working in healthcare.

The process of identifying potential participants to meet the study inclusion criteria was discussed amongst all authors and this approach produced a list of 46 names who were to be sent the e-survey. Of the 46 emails sent in early June 2017 inviting completion of the e-survey, a number were immediately removed due to email addresses registering as being ‘unknown’ or having an ‘out of office’ bounce-back notice for the duration of the period of data collection. This left 31 potential participants who were asked to respond within 3 weeks of receiving the e-survey request email and link to the e-survey. This time period was decided upon taking into account participant’s workloads and the possibility of participants being on annual leave, balanced with the timescales of the study. One reminder email was sent after a period of 2 weeks. In total, 19 completed e-surveys were returned by the end of the period of data collection which represented a 61% response rate. As seen in Table 1, 84% of the sample indicated that they had been working in dementia care for more than 16 years and 15 of the 19 participants indicated they had had some clinical experience of a dementia assessment ward.

**Table 1.** Sample frame: UK National dementia leaders.

Role profile and qualifications (where stated)	Role Domain(s)	Years’ experience
Academic 1 (General practitioner)	Research/Education	16–20
Academic 2 (Psychiatrist)	Clinical/Research/Education/Policy & Guidance	20+
Academic 3 (Geriatrician)	Research	20+
Academic 4 (Occupational therapist)	Research	20+
Academic 5 (Consultant Psychiatrist)	Clinical/Research	20+
Academic-6 (RMN and RGN)	Research/Education	16–20
Academic-7 (Social worker)	Education	16–20
Clinical psychologist	Clinical	16–20
Consultant Geriatrician 1	Clinical/Education	11–15
Consultant Geriatrician 2	Service Provision & Development	20+
Consultant Physician	Clinical/Research/Education/Policy & Guidance	16–20
Person living with dementia (medical background)	Education	6–10
Senior nurse 1 (RMN)	Clinical	6–10
Senior nurse 2 (RMN)	Clinical/Research/Education/Policy & Guidance	20 +
Senior nurse 3 (RMN and RGN)	Clinical/Education/Strategic	20+
Senior nurse 4 (RMN)	Clinical/Education/Policy & Guidance	20+
Senior nurse 5 (RMN)	Clinical/Education/Policy & Guidance	16–20
Senior nurse 6 (RMN)	Service Development/Strategy/Research/Education	20+
Speech and Language therapist	Clinical/Research	20+

Phase 2 of data collection involved 20 semi-structured interviews with clinical staff from Daisy and Jasmine Wards. Inclusion criteria for phase 2 were twofold:

 (i) Staff working in a clinical role on Daisy and Jasmine Wards or the older adult community mental health team involved in that setting, including registered nurses, social workers, psychologists, allied health professionals, nursing assistants/support workers.
 (ii) An ability to communicate in English.

Informed by the iterative nature of the study design, and the lead author’s clinical experience and sensitivity, the semi-structured interview guide was framed around four main questions:

 (i) What factors contribute towards a person with dementia on the ward being positioned as complex?
 (ii) Can you think of a patient you have cared for recently with dementia who you felt was complex and describe what made them complex?
 (iii) How do you assess and intervene with someone on the ward who is complex?
 (iv) If a patient on the ward is complex, or has complex needs, how and where would this be reflected within the care records?

In phase 2, prior to participant recruitment, the first author undertook a number of awareness raising sessions about the study with staff on both Daisy and Jasmine Wards. All in-person interviews were subsequently undertaken within the hospital building, but not on the wards themselves. Table 2 outlines the dementia assessment ward staff members who took part in the interviews.

**Table 2.** Phase 2 - participant sample and coding frame: Dementia assessment ward practitioners.

Dementia practitioners			
Participants role and study code	Ward	Years qualified	Time in dementia care
Allied health professional 1	Daisy	8 years	9 months
Allied health professional 2	Jasmine	33 years	30 years
Allied health professional 3	Jasmine	12 years	3 years
Consultant Psychiatrist 1	Daisy	Consultant for 11 years	14 years
Consultant Psychiatrist 2	Jasmine	Consultant for 4 years	8 years
Deputy ward manger 1	Daisy	3 years	12 years
Deputy ward manager 2	Jasmine	32 years	5 months
Nursing assistant 1	Daisy	16 years	16 years
Nursing assistant 2	Daisy	N/A	14 years
Nursing assistant 3	Daisy	N/A	21 years
Nursing assistant 4	Daisy	N/A	2 years
Psychologist	Jasmine	11 years	11 years
Registered General Nurse	Jasmine	38 years	7 years
Senior nursing practitioner	Jasmine	32 years	30 years
Staff nurse 1	Daisy	6 months	6 months
Staff nurse 2	Daisy	12 years	14 years
Staff nurse 3	Jasmine	2 years	18 years
Staff nurse 4	Jasmine	10 years	20 years
Ward manager 1	Daisy	6 years	6 years
Ward manager 2	Jasmine	32 years	5 months

Continuing in phase 2 of the study design, Table 3 outlines membership of the conducted focus group with community staff who admitted people with dementia to either Daisy or Jasmine Wards.

**Table 3.** Phase 2–participant sample frame: Community practitioners.

Community practitioners		
Participants role and study code	Years in community team	Years in dementia care
Community psychiatric nurse 1	9	30
Community psychiatric nurse 2	10	10+
Community psychiatric nurse 3	17	30+
Community psychiatric nurse 4	6	6
Community psychiatric nurse 5	1	8
Community psychiatric nurse 6	7	7+
Occupational therapist	13	13
Social worker 1	10	Did not say
Social worker 2	20+	30+

The questions asked in this phase of the study were framed around:

 (i) The reason(s) why a person with dementia is usually admitted to a dementia assessment ward.
 (ii) The situation at home at the time of admission and if, and how, it was described as being complex.
 (iii) The community staffs’ expectations of the admission to the dementia assessment ward.
 (iv) If the community staff had been involved in the assessment process on the dementia assessment ward and in the discharge process.
 (v) How a compulsory [i.e. use of a detaining section under the Mental Health Act 1983] admission to the dementia assessment ward for the person with dementia was managed and experienced.

In phase 3, a case study approach (Yin, 2014) was assimilated into the interpretive description design (Thorne, 2008). Undertaking a series of case studies using a mixed qualitative methods approach (i.e. interviews, observation and documentary analysis) enabled a more detailed exploration of how complexity presented in a person with dementia who was resident on either Daisy or Jasmine Wards. In terms of personnel, an individual case comprised of: a person living with dementia resident on either Daisy or Jasmine Wards; their visiting family carer; the named clinician on the ward involved in that person’s care; and a care record review. The inclusion criteria for people with dementia resident on the ward to be approached about participation were threefold:

 (i) They had been an inpatient for a minimum of five days.
 (ii) The care team had to have commenced their assessment and care interventions at the time of recruitment.
 (iii) The person with dementia was regarded as ‘complex’ in their presentation by the care staff.

Consent was obtained to both observe and interview people living with dementia about their feelings/experiences of being on either of the recruited Wards. The interview guide for patients looked to explore:

• How the person with dementia understood their admission.
• How the person understood their dementia.
• What complexity meant to the person with dementia.
• How the person with dementia understood their detainment on the dementia assessment ward.

As shown in Table 4, four people with dementia who were resident on either Daisy or Jasmine Ward were recruited into the study together with their main family carer and also the named clinician responsible for their care.

**Table 4.** Phase 3–Case Study Sample Frame: People with Dementia, Family Carers, Key Worker and Care Record Review

Case	Diagnosis	Age	Legal status	Personal consultee	Interviews	Periods of observation (in hours)	Care record review record
1.Brian	Alzheimer’s disease	80	Section 3 Mental Health Act	Wife Gloria	Wife Gloria Deputy ward manager Joan	11	Yes
2.Eric	Vascular dementia	78	Section 3 Mental Health Act	Wife Anna	Wife Anna Consultant psychiatrist	2	Yes
3.Charlotte	Young onset Alzheimer’s disease	61	Section 3 Mental Health Act	Husband Harry	Husband Harry Named nurse Linda	8	Yes
4.Celia	Probable mixed Vascular dementia/Alzheimer’s disease with psychosis	82	Section 3 Mental Health Act	Husband George	Husband George Occupational therapist Lucy	7	Yes

All four participants with dementia were assessed as lacking the capacity to consent and therefore required personal consultee consent to take part in the study, in line with the Mental Capacity Act (Department of Health, 2005). The lead author of this article undertook all the ethnographic observations of people living with dementia that were part of the phase 3 case study design and mixed methods approach. All periods of observation on either Daisy or Jasmine Wards were approached in a way that was minimally disruptive to everyday activities and field notes were captured in a notebook as events happened in real time or were reflected upon shortly after the event. All notebook entries were dated. In order to protect participant’s dignity, a decision was made not to undertake observations during the provision of direct care which required privacy, e.g. washing, dressing, bathing, and using the toilet. In addition, prior to undertaking periods of observation, permission was gained from the care team and the person’s personal consultee. All staff on Daisy and Jasmine Wards received on-going study awareness raising sessions by the first author about what the observations would entail and their purpose. A total of 28 h of observation was conducted across the four participants.

Each of the personal consultees named in Table 4 became the main family carer recruited into the study. The interview guide for family carers explored:

• The family carer’s understanding of their relative’s admission to the dementia assessment ward and their expectations of the admission.
• The situation at home prior to the admission being arranged and the process of their relative’s compulsory assessment.
• How the family carers understood complexity in their lives.
• The experiences of visiting their relative on the dementia assessment ward.

For the named clinician on the ward involved in that person with dementia’s care, the interview schedule followed the parameters of the previously shared phase 2 semi-structured interview schedule with clinical staff from Daisy and Jasmine Wards, but this time the questions were able to be personalised to the individual concerned.

For the four people with dementia who took part in the study, permission was also granted to undertake a care record review so that a documentary analysis of the clinical notes could form part of the developed case study.

### Data analysis

Data analysis followed the approach recommended by Thorne (2014) with the first author constantly reflecting and asking questions of the data such as: ‘Why is this here?’, ‘Why not something else?’, ‘What does this mean?’ (Thorne, 2013). The parameters of the emergent practice model were then tested out and refined through dialogue with all other members of the authorship and the study advisory group. In this approach to data analysis, Thorne (2013) discourages the use of data sorting software at the start of data analysis and, instead, the researcher/research team are encouraged to work manually by constantly reflecting, examining and challenging the data until they are formed into interlinked and interconnecting parts that tell the researcher/research team something that was not known before. This constant pushing and pulling of the data continues until a ‘tentative truth claim’ (Thorne, 2014, p.7) is established that describes what is common within the clinical phenomenon under study: in our case, this related to the meaning and practice of complexity in dementia on a dementia assessment ward.

A focus on clinical relevance is therefore a purposeful intention of an interpretive description study and resulted in the development of the ‘3 Fs Model of Complexity’. As we will shortly explain, the 3‘Fs’ in the model stand for ‘Fixed’, ‘Flexible’ and ‘Fluctuating’ domains of complexity with the ‘Fixed’ and ‘Flexible’ domains each having identified and interconnecting components. All domains and components are further supported by qualitative text in the Supplemental Material.

## Findings

Before outlining the ‘3 Fs Model of Complexity’, it is important for us to state that there was consensus amongst all study participants across all phases of the study that only people with dementia who were complex in their presentation should be admitted to a dementia assessment ward. Data from all phases of the study revealed that such complexity is something about being ‘above or outside the norm’ for what would be expected at that stage and that time in the person’s presentation of dementia. Amongst study participants operating clinically, there was also a view that adopting the term ‘complexity’ in practice is a helpful way of bringing together a number of components and their interaction with one another, as opposed to simply recording the person with dementia’s main behaviour, problem, intervention or symptom that was seen at that moment in time.

### Fixed domain of complexity

The Fixed domain of complexity is comprised of six components which have the ability to interact with one another. Each of these six components are ‘fixed’ in the sense that they are *always present* to varying degrees for a person with dementia to be seen and assessed as being complex. The numbering of these six components is not intended to be hierarchical.

**Component 1) Presence of dementia:** As perhaps expected, the diagnosis and presence of dementia was seen to be a ‘Fixed’ component of complexity. However, that said, continually diminishing memory performance and complications arising from this aspect of a person with dementia’s cognition was repeatedly highlighted in the data set as a feature that increased complexity owing to its enduring impact on the individual and its deleterious effect on personal agency. Moreover, whilst complexity was reported as being seen at any stage in the presence of dementia, participants most often attributed it to its moderate and advanced stages, especially when the dementia was seen to progress and deteriorate rapidly. Whilst all types of dementia were highlighted as having the potential to be complex, three types of dementia were signalled out as being prone to be especially complex, these were: Lewy body dementia, fronto-temporal dementia and young onset dementia.

**Component 2) Life story:** This component refers to the introduction and assimilation of the narrative of ‘dementia’ into the person’s present and future life story and its impact on the person’s identity and sense of self. However, participants stressed that the person with dementia re-living and re-experiencing past life traumas in the present significantly contributed towards a presentation of complexity. In addition, the intersection of significant life events on a person with dementia’s developed personality traits, and how those personality traits subsequently reacted and responded to events in the present, were also seen as important contributory factors to complexity.

**Component 3) Impaired communication:** In this context, impaired communication refers to a person with dementia being unable to initiate and respond to conversation in a way that others can understand and to receive and process communication from others. This aspect of complexity also encompasses a person with dementia’s inability to verbalise or embody personal thoughts and feelings and to have an on-going struggle to make personal needs and wants known to others.

**Component 4) Reduced insight and capacity:** Drawing attention to dementia as a progressive condition which undermines personal decision-making, participants linked the person with dementia’s reduced levels of insight and capacity to complexity. Issues of reduced insight and capacity were also associated with the difficulties that this created when a person with dementia required supportive and/or care interventions. Moreover, any degree of hallucinations experienced by the person with dementia, and the risks posed to the person him- or herself and others during this episode of lived experience, was also seen to contribute significantly towards a presentation of complexity.

**Component 5) Changed behaviour:** Numerous illustrations and examples were shared across all phases of the study to describe the changed behaviour(s) which led to a person with dementia to be seen as being complex on a dementia assessment ward; the most commonly described behaviours were: agitation, aggression, violence, physically resisting personal care interventions, intrusiveness, shouting, screaming, continence problems and throwing oneself to the floor. These descriptions of changed behaviours do not differ from those that are well-documented in the literature (see for example: James & Jackman, 2017). However, it was not simply the presence of such changed behaviour(s) that participants stated was a feature of complexity, but their severity, duration (rapid or enduring), degree of unpredictability and receptiveness, or otherwise, to a supportive intervention.

**Component 6) Risk:** The existence of risk was seen as a key indicator for the person with dementia’s compulsory admission to a dementia assessment ward and four key areas of risk were identified that were associated with this outcome: i) the person with dementia’s willingness to accept help; ii) the risk of harm to the person with dementia and/or to others; iii) risk stemming from the person with dementia’s multiple needs; and iv) the person with dementia being unable to maintain safety at home/in the community. In addition, high levels of risk featured as a consequence of other identified components in the Fixed and Flexible domains of complexity.

### Flexible domain of complexity

The Flexible domain of complexity is comprised of 14 components, but unlike the six components in the ‘Fixed’ domain, not all phases of the study identified all the reported components. In the ‘3 Fs Model of Complexity’, should they be present, any one, or any combination, of the 14 components in the Flexible domain of complexity will *always interact* with the 6 components in the Fixed domain of complexity. The extent of interaction will vary and they may also *interact with other components in the Flexible domain*. Importantly, these 14 components are ‘flexible’ in the sense that a person’s presentation of dementia may be complex *without any one, or any combination, of these components being present*. Moreover, any one, or any combination, of the 14 Flexible components of complexity may commence, end and/or re-commence again over the course of a person’s dementia and their intensity may vary during their occurrence. In our ‘3Fs Model of Complexity’, this variation in intensity is termed ‘Fluctuating’ and is the final ‘F’ in the ‘3 Fs Model of Complexity’. We will return to the Fluctuating domain after outlining each of the 14 components of the Flexible domain of complexity and again, the numbering is not intended to be hierarchical.

**Component 1) Physical health conditions:** This component concerns itself with the presence of acute and/or chronic co-morbid physical health conditions. Physical health conditions which were frequently reported in the data and aligned to the person with dementia on the dementia assessment ward included: diabetes, urinary tract infections, chest infections, atrial fibrillation, renal impairment, arthritis, chronic obstructive pulmonary disease, cancer and coronary heart disease. Attention was specifically drawn to the effect of physical health conditions on a person with dementia’s memory and cognition (Fixed component 1) and reduced insight and capacity (Fixed component 4).

**Component 2) Pain:** Pain was described as being under-recognised and under-reported by people with dementia, families and care staff and that under-treated pain was a significant practice issue on a dementia assessment ward. The presence of pain was frequently associated with ‘impaired communication’ (Fixed component 3) and the person with dementia’s inability to report pain; this was also reported as an overlap between the effect of pain and its impact on ‘changed behaviour’ (Fixed component 5).

**Component 3) Mental health problems:** In the data, this term was used to cover a range of conditions that ranged from anxiety to psychosis. In particular, the person with dementia’s experience of hallucinations, depression and delusional thoughts were linked to complexity.

**Component 4) Diet and fluid changes:** Issues attributed to this component were numerous and for people with dementia included: the effect of not taking enough diet and fluids and the subsequent increased risk of dehydration, malnutrition and/or infection; forgetting how to place food to the mouth; not recognising food; and people with dementia just wanting to eat sweet foods all the time and therefore experiencing a poor diet.

**Component 5) Impaired self-care ability:** This component relates to the person with dementia’s inability to maintain their own self-care and hygiene needs. For example, participants identified that people with dementia may no longer be able to physically perform tasks such as washing, showering and changing clothes, or that they no longer realised that these elements of self-care needed to be undertaken. In addition, it was acknowledged that people with dementia may also experience continence problems in that they may no longer be able to recognise the need to go to the toilet; that they may no longer remember where the toilet is located; they may no longer be able to recognise a toilet as a toilet or they may not realise that they had been incontinent.

**Component 6) Sensory impairment:** The presence of a sensory impairment (i.e. sight, hearing, smell, taste and touch) was raised across the data set, but more so in phases 2 and 3 of the study design when the sensory impairment intersected with ‘impaired communication’ (Fixed component 3), such as the person with dementia being unable to hear and therefore being unable to communicate effectively.

**Component 7) Mobility changes:** Discussions around mobility changes were used by participants to describe: unsteadiness, requirement of walking aids, the ability to negotiate changes to floor levels, mobilising around obstacles such as chairs, tables and doors, and the increased risk of falls which occurs when a person’s mobility deteriorates. In phase 2 of the study especially, mobility changes featured most strongly as a component of complexity with practitioners closely linking mobility changes to an increased risk of falls.

**Component 8) Sleep changes:** These words were mainly used to describe the person with dementia having a poor sleep pattern, or being unable to sleep at all during the night. There was also awareness that this change in sleep pattern can also be interrelated with the person’s life story as seen in component 2 of the Fixed domain of complexity. There was also an awareness of the risks that can occur when a person with dementia does not get enough sleep and how these can affect other ‘Fixed’ and ‘Flexible’ components of complexity.

**Component 9) Frailty:** In the main, people living with frailty will not recover as quickly after an illness, accident or stressful event. This prospect was highlighted by participants in some phases of the study through an understanding that a number of people with dementia were at an increased risk of losing their in-built reserves because of the progressive nature of dementia and living with (any) other physical healthcare needs.

**Component 10) Swallowing difficulties:** Although not frequently represented in the data, participants acknowledged that swallowing difficulties become more commonplace as a dementia progresses and can be caused by the progression of the dementia. In phase 2 of the study especially, practitioners talked about swallowing difficulties contributing to complexity when, for example, they prevented a person with dementia from being able to take oral medication or contributed towards episodes of choking. It was also pointed out that if swallowing difficulties were overlooked, the person with dementia was at risk of weight loss, malnutrition, dehydration, choking and aspirational pneumonia.

**Component 11) Medication issues:** Here, the data identified polypharmacy, compliance, drug sensitivities, side-effects and covert medication administration as areas where complexity could arise if this aspect of care practice was not acknowledged or assessed.

**Component 12) Environmental effects:** This component concerns the effect the environment can have on people with dementia resident on the dementia assessment ward. For some people, it was the effect of the physical environment that contributed towards complexity, such as the unfamiliar layout, lighting, heating and locked doors whilst for others, it could be living in such close proximity to other people in an unfamiliar and shared environment. Study participants also shared that for some people with dementia, an admission into hospital could also cause deterioration in their dementia and add to their complexity through separation from a familiar home environment and attachment figures.

**Component 13) Family involvement:** This may, at first, seem a contentious component of complexity as in most cases family members are supportive and want what is best for the person with dementia. However, comments about negative family involvement, such as denial about the diagnosis, or conflict with care management decisions and experiences of ‘difficult’ families visiting the dementia assessment ward, were visible throughout the data set. Whilst this will need careful assessment and recording, viewing complexity through a family systems lens may be a useful way of thinking about and responding to this identified component.

**Component 14) Attitude and approach of others:** This final component concerns itself with how a carer, be that professional or family, may fail to connect, engage, communicate and deliver care to the person with dementia. Often, this ‘failure’ may stem from a lack of awareness and understanding about dementia and how it affects the person living with the condition. As such, this contribution towards complexity rests not with the person with the dementia him- or herself, but with those in a position of responsibility and care - as also reflected in Flexible component 13.

### Fluctuating domain of complexity

As shared earlier, the third ‘F’ on the ‘3 Fs Model of Complexity’ stands for Fluctuating. This means that the components of both the Fixed and Flexible domains of complexity are not static and their presentation can change with acuity and over time. The data indicates that Fluctuating occurs in the Fixed and Flexible domains of complexity for three main reasons: i) when something changes in an existing component in either the Fixed and Flexible domains; ii) when an additional ‘new’ Flexible component of complexity comes into play; and iii) in response to an intervention. Any of these reasons can result in an increase in acuity and for a person with dementia to become more complex in their presentation.

**Figure 2.**
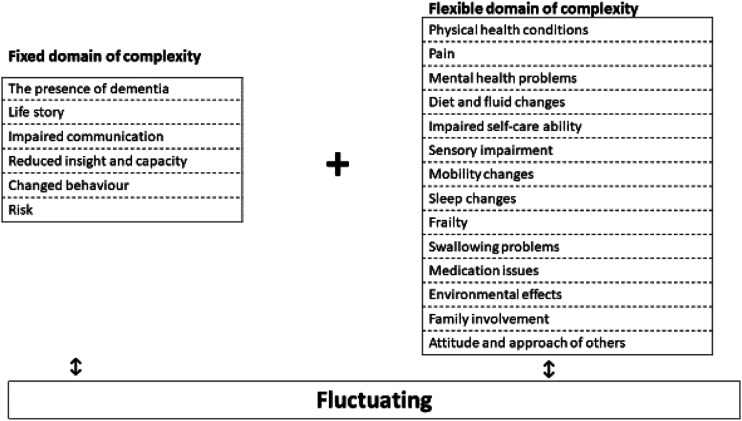
The integrated and interactional properties of the 3fs model of complexity.

When integrated, it becomes possible to plot/map the interaction between the Fixed, Flexible and Fluctuating domains of complexity and for this integration to provide a more complete picture of assessment for the person with dementia at any given moment of time. This integrated and interactional process is represented in Figure 2.

Figure 2 exists to be further refined, adapted and tested in clinical practice and, as such, it addresses what Thorne (2008) called the generation of ‘useable knowledge’ (p.16) in the application of interpretive description.

## Discussion

In outlining the properties and components of the ‘3 Fs Model of Complexity’, this study has offered a timely opportunity to provide some emergent clarity to the construct of complexity and dementia assessment ward practice. In many ways, our study supports the earlier findings of McGeorge (2010) on this topic area but has extended them by identifying ‘Fixed’ and ‘Flexible’ components of complexity and outlining an interactive practice model that could be further tested, refined and used in everyday clinical practice. As an illustration, the ‘3 Fs Model of Complexity’ could help to guide the selection of tailored and personalised interventions for people with dementia on a dementia assessment ward and act as the basis for developing and implementing formulation approaches, which is increasing in popularity in both the literature and practice (Holle et al., 2017; Jackman & Beatty, 2015; James et al., 2021). In this latter context, formulation for people with dementia consists of four phases: i) a description of the observed behaviour, ii) analysis of the causes of behaviour, iii) identification of potential interventions and iv) an on-going review of efficacy (Holle et al., 2017). In order to analyse the cause of the presenting changed behaviour(s), a detailed assessment of the person with dementia is required and this assessment includes, for example, details of a person’s life history, pre-morbid personality, cognition, physical health, and social situation (Duffy, 2016; Jackman & Beatty, 2015). The ‘3 Fs Model of Complexity’ has the potential to support formulation approaches as it outlines all the components of a comprehensive assessment and serves as the bio-psycho-social-physical framework needed from which staff could identify the often multiple interlinked causes of changed behaviour(s) and the context in which they occur (see also: Keady et al., 2013). Without the provision of a comprehensive assessment to facilitate this understanding as to the causes of behaviour, identified interventions may not lead to a reduction, or removal, of the seen and experienced behaviour. Furthermore, the ‘3 Fs Model of Complexity’ could have applicability outside of the dementia assessment ward from where it was generated and be used in formulation led interventions developed for use in domestic and care home settings, for example (Holle et al., 2017).

To date, in the UK, a discourse on complexity and care on a dementia assessment ward has largely been omitted from all levels of dementia care practice, planning, research and policy-making. Whilst there may be a number of reasons for this, including the challenge of accessing these sites as areas of research attention, one of the major stumbling blocks we encountered in conducting this study was in the time it took to prepare the forms to obtain Health Research Authority ethical permission to involve people with dementia in research who are under a section of the Mental Health Act (Department of Health, 1983; and see Table 4), and thereby lacked the capacity to consent. The amount of documentation and protocols necessary to be generated and approved to do this type of work in the NHS was significant and, if we are being honest, more than a little daunting. Whilst there is no easy solution to this dilemma given the legal safeguards that exist to protect people with dementia in such a vulnerable situation (Department of Health, 2005), without an opportunity to more readily involve people with dementia in research who are on the receiving end of such legal safeguards, those voices will remain absent from the literature and the debate. In our opinion, that is iniquitous as whilst diminished capacity and increased complexity for people with dementia may go hand-in-hand, without a focus on this aspect of lived experience in the trajectory of dementia, research in the field will continue to be skewed towards the earlier stages of dementia where consent can be more readily ascertained, validated and recorded. A broader research and policy direction is called for.

Linked directly to the preceding paragraph, the discourse around complexity and dementia shared in this article, and in the Supplemental Material, inverts the dominant ‘living well with dementia’ narrative that has permeated the dementia studies field well for over a decade (see for example: Department of Health, 2009; Quinn et al., 2021). People with dementia in our study were plainly not living well with dementia and more attention needs to be paid to a more inclusive agenda that does not discriminate, stigmatise or ‘other’ those individuals - and families - who experience the more complex and challenging face of dementia. In taking up this challenge, Bartlett et al. (2017) have suggested that more space needs to be created in the dementia studies field to accommodate the narrative of ‘suffering with dementia’ and that future actions should be taken on the basis of ‘actualities and evidence rather than presumption and sentiment’ (p.177). Our study would certainly reinforce this messaging and the need for a more inclusive public narrative in dementia.

One area that could help in the normalisation of such complex experiences is in the conduct of life story work. Listening to people with dementia and understanding their stories is now seen as an essential component of ‘good’ dementia care practice (Gridley, et al., 2016; Kindell et al., 2014) and as seen in this study in the shape of ‘Fixed’ component 2. Knowledge about an individual’s life story can help change staff member’s views about a person with dementia and help to see the ‘person’ beyond the changed behaviour(s) (McKeown, et al., 2010). However, as we encountered in phase 3 of this study, there was little, if any, information about the life stories of each of the four case studies contained in their separate care records. Use of the ‘3 Fs Model of Complexity’ could therefore be helpful in providing a structure to care staff so that the person with dementia’s identity and selfhood is maintained and uppermost in the thoughts and actions of practitioners during a period of admission to a dementia assessment ward.

## Study limitations

There are three main study limitations. First, the online electronic survey undertaken in phase 1 of the study had only 19 responders; a larger response rate may have provided greater insights into how dementia leaders position and construct complexity in dementia. Second, data collection in phases 2 and 3 of the study occurred in one NHS Mental Health Trust in the north of England. The involvement of other NHS Mental Health Trusts from across the UK may have yielded more comparative data and further developed the concept of complexity across clinical settings in different geographical areas. Third, whilst this is the first study of its kind that has explored the concept of complexity from within the context of dementia assessment ward practice, the ‘3 Fs Model of Complexity’ requires additional research and clinical use to further refine and test its applicability and utility.

## Conclusion

The study has enabled a practice model about the presentation of complexity in the dementia assessment ward to be inductively and iteratively developed and reported. With further testing and refinement, the ‘3 Fs Model of Complexity’ has the potential to become an assessment framework for people with dementia admitted to such environments and could help to guide the development of personalised interventions, including formulation approaches. Our study findings also support the positioning that complexity for people with dementia has bio-psycho-social-physical origins and that it is a fluid, unpredictable and dynamic state. This study has also established the importance of the interaction of components within and between the Fixed and Flexible domains in the presentation of complexity and the importance of accounting for acuity. To develop the field in the future, more attention needs to be paid to the dementia assessment ward as a site for research and to the lived experience of people with dementia without capacity. Without such a focus, the status quo will prevail and the experiences of the most vulnerable people living with dementia, silenced.

## Supplemental Material

Supplemental Material - Introducing the ‘3 Fs model of complexity’ for people with dementia accessing a NHS mental health inpatient dementia assessment ward: An interpretive description study Click here for additional data file. Supplemental Material for Introducing the ‘3 Fs model of complexity’ for people with dementia accessing a NHS mental health inpatient dementia assessment ward: An interpretive description study by Lesley Jones, Nicky Cullum, Ruth Watson and John Keady in Dementia
