# Supplementary material for: Introducing the ‘3 Fs model of complexity’ for people with dementia accessing a NHS mental health inpatient dementia assessment ward: An interpretive description study
Source: Dementia (London). 2022 Nov 8;22(1):85–104. doi: 10.1177/14713012221136313 (PMC9772894; doi:10.1177/14713012221136313)
Supplement: Supplemental Material - Introducing the ‘3 Fs model of complexity’ for people with dementia accessing a NHS mental health inpatient dementia assessment ward: An interpretive description study [file sj-pdf-1-dem-10.1177_14713012221136313.pdf]

Supplemental Material

Selection of quotes from across the data set to substantiate the meaning and construction of complexity and each domain component

Meaning and construction of complexity

Phase 1	'I think complexity on the dementia assessment ward is very dynamic and it moves all the time; so it is in waves, I suppose. Waves that can get higher and higher.'	Senior Nurse 2
Phase 2	'Complexity is about all factors happening in conjunction with each other and you have the patient in the middle, you can't put any of the factors in silos, can't just look at one of the areas.'	Allied Health Professional 3
Phase 2	'When I think of complex I think of the whole person, you know what it is that makes them complex.'	Occupational Therapist
Phase 2	'Probably for me, complexity would be in the amount of interventions we have to do to for that person to remain alive. For instance, if we didn't feed them, get them out of their bed, change them when they're soaking wet or doubly incontinent. We can't just leave them where they were or not give them a drink, or give them the medication that they require to remain pain-free. We need to manage whatever breakdown needs assisting with at that time. That is the higher end of complexity to me.'	Ward Manager 1
Phase 3	'You can't be rational. There is no rational in it at all. Its complex because you can't apply normal logic to something that isn't normal. Because the illness isn't normal and, for me, and I suppose for everybody, it was a learning curve, trying to understand what was going on. And yes its complex.'	Relative interview with George (case study 4)

Fixed domain of complexity

Component 1: Presence of dementia

Phase 1	'There is no complexity without the presence of dementia'.	Senior Nurse 6
Phase 2	'Being complex is about being an above average patient, in the sense that you would have a diagnostic category and a complex person. In	Consultant Psychiatrist 1

	other words, it is someone who is above average in their presentation within that diagnostic category of dementia'.	
Phase 3	The types of dementia and how it affected the person was often not documented in care plans and risk management plans.	Care record analysis

Component 2: Life story

Phase 1	'It's about things in the person's past that may be relevant; for example, adults who have experienced trauma or distressing care as a child may find a residential or hospital setting difficult'.	Speech and Language Therapist
Phase 2	'Everything that been before affects who we are now and how we cope'.	Social Worker 2
Phase 2	'You couldn't get her on to the toilet, because all of a sudden, when you wanted to help her get on, she was fighting for her life. And then it ended up two, maybe three having to do that for her. And it was horrendous really for her and not pleasant for staff either. But then you learn after that this lady was raped when she was younger. So she's genuinely fighting for her life. So how must she feel?'	Ward Manager 1
Phase 3	Celia (case study 4) was described by her husband George as being 'strong, fiery and independent' and someone that, when frightened, would 'get angry'. These are all traits that were clearly evident every time Celia was observed on the dementia assessment ward. Celia had also spent most of her life looking after others; she came from a large family, had brought up a number of her siblings and, within her own family, had been the matriarch and head of the family. These were all roles that she attempted to recreate/continue within the ward environment and she would become angry if she believed this was being challenged in any way.	Case study: observation and interview

Component 3: Impaired communication

Phase 1	'I see complexity on the ward as through a range of dementia communication problems'.	Academic 4
Phase 2	'The biggest thing I have seen on the ward is communication. So when you're talking to someone with dementia, but they can't understand what you're saying, and you can see the frustration in their face, a lot of complexity	Staff Nurse 1

	derives from that'.	
Phase 2	'It's around the fact that they can't tell you when they are hungry, when they need to go to the toilet, when they want something, when they are in pain or they just want to ask a question and they can't communicate their needs to you, or anyone else. And if English isn't the person's first language, it makes it all so much harder.'	Deputy Ward Manager 2
Phase 3	Charlotte was still able to verbalise occasional words and neologisms, her ability to blaspheme had been retained, she could repeat one of two words that she could hear and she could still make verbal noises. However, what Charlotte was no longer able to do, was symptom report or explain to staff, or her family, her thoughts, feelings, concerns or needs in any way. Nor was she able to engage in a meaningful conversation, initiate a conversation or give any indication she could comprehend or process information that was being given to her.	Case study observation

Component 4: Reduced insight and capacity

Phase 1	'What makes it complex is a lack of insight by the person with dementia a lack of decision-making capacity'.	Academic 5
Phase 2	'The typical patients that are admitted to [name of the ward] as a result of the dementia severity do not understand that they have an illness, they do not understand they need to come to hospital for assessment and treatment at all.'	Community Psychiatric Nurse 4
Phase 2	'I mean, if somebody has got no insight into their needs, they have no insight that they actually need help. Maybe, they haven't got the insight to realise that they need help with personal care, or they need help to go to the toilet and you will get, 'No, I'm fine, I can do it myself' and I will say, 'Oh, no, I need to give you some help'. But, quite often, they don't take that advice kindly, and that can cause agitation, aggression, and resistiveness.'	Staff Nurse 3
Phase 2	'It's very hard to get through to someone that, you know someone that's not got any real kind of capacity into their illness to explain to them that, you know, what you're actually smelling or seeing isn't necessarily real.'	Nursing Assistant 4
Phase 2	'We have doctors that come onto the ward that's never worked in a dementia assessment ward and they wouldn't examine a patient because the	Consultant Psychiatrist 2

	patient is refusing, but the patient doesn't have capacity and they're so desperately acutely unwell, you need to be examining that patient in their best interest. If you leave them, the chances are that they will die because of an acute problem.'	
Phase 3	Brian, Eric, Charlotte and Celia were all detained in hospital on sections of the Mental Health Act 1983 as they required assessment and treatment, but due to impaired insight and capacity were not willing to be in hospital voluntarily. The care records also indicated that these individuals did not have the capacity to make a decision about, for example, being in hospital, accepting medication and treatment and their future care needs.	Observation and care record review

Component 5: Changed behaviour

Phase 1	'What I have seen on the ward and is complex is shouting, screaming, throwing oneself on the floor and refusal of care.'	Senior Nurse 4
Phase 2	'Now the level of aggression we're talking is out of the ordinary, not what the people…the average patients with that type of dementia will experience. So that would be another characteristic of complexity in this gentleman. The aggression was very intense and unpredictable and happened so many times a day. So right from when the gentleman wakes up, you just don't know what to expect.'	Consultant Psychiatrist 1
Phase 2	'Well … I suppose it challenging when we have tried lots and lots of different type of interventions to make a difference and nothing seems to work. Then we get to the point where the staff team go, 'What else can we do, where can we go with this?''	Deputy Ward Manger 1
Phase 3	Charlotte displayed changed behaviours that were severe, unpredictable and did not have easy solutions. During my observations of Charlotte she had increasing periods of vocalisation and she would shout and swear loudly. The reasons for this were not obvious. In addition, I did not read in Charlotte's clinical record any identified possible causes for these behaviours nor any guidance about care interventions. It was also apparent on observation that clinical staff were unsure at times about how to deal with her presentation. Indeed, I observed a number of	Observation

	different approaches being used, ranging from ignoring her, trying to ascertain if she had an unmet need to removing her from the day area.	

Component 6: Risk

Phase 1	‘Complexity on the ward is about high levels of risk which the person does not appreciate or accept the help of others in the management of these risks.’	Senior Nurse 1
Phase 2	‘So when we are looking at complexity, we are mainly looking at risk. You know, you are looking at risk to self, risk to others and risk to health.’	Psychologist
Phase 2	‘He has actually needed to be on one-to-one observation because of his risk of falling and also his impulsive behaviour at times where he gets an idea that somebody’s against him and he will engage that person in a fight. So he has risks that somebody else could become agitated and retaliate to him as well.’	Allied Health Professional 3
Phase 2	‘If you can imagine complexity as a graph then people with dementia admitted to a dementia assessment ward are at the top of the graph with multiple needs, no capacity and high levels of risk.’	Community Psychiatric Nurse 4
Phase 3	The presence of high levels of risk was evident in all four case studies. The case of Eric demonstrated how risk can change and evolve as a dementia progresses. When Eric was initially admitted he was aggressive and violent, he required covert medication and his mobility was poor. These three factors alone made him a risk of harm to others, risk of his physical and mental health deteriorating if he would not take prescribed medication, and a risk of falls due to poor mobility. These factors were also compounded by the presence of dementia, changes to his cognition and impaired insight and capacity. Within a short period of time, Eric’s dementia had progressed to the terminal phase and, as such, his risks had changed and evolved as he was approaching end of life. He was now at risk of pressure areas developing due to reduced mobility, risk of developing chest infection due to immobility and at risk of attack from other more intrusive patients.	Observation and interviews with care staff and relatives

Flexible domain of complexity

Component 1: Physical health conditions

Phase 1	'Multiple physical conditions and their interaction with the presence of dementia.'	Academic 2
Phase 2	'It's the physical health problems that people have and most of the patients on a dementia ward have physical health problems.'	Allied Health Professional 2
Phase 2	'Sometimes we see people with cancers and sometimes we have to diagnose the cancers, because they come to us and they deteriorate suddenly on the ward and then we examine then and we find things. So that, kind of, may mask some symptoms or may have exacerbated some symptoms'.	Consultant Psychiatrist 2
Phase 3	Three of the case studies had presenting physical health issues with Eric (case study 2) demonstrating how his complexity had changed from a focus upon his changed behaviour to meeting his physical health care needs when he was approaching end of life.	Lead author/researcher notebook entry

Component 2: Pain

Phase 1	'Under-recognised and under-treated pain is a huge issue in my work on the dementia assessment ward'.	Senior Nurse 3
Phase 2	'There is a definite difference in his presentation when he is in pain. He does become more agitated and hostile, threatening as well I suppose. He can be difficult to manage. It can take three to four staff to deliver his personal care, but a lot of the time it can be down to pain.'	Staff Nurse 1
Phase 3	The presence and effect of pain on the cases studies was perhaps a little more subtle and not immediately obvious. In case study 4, Celia was observed to be limping on numerous occasions and the care team thought she may have had arthritis. However, Celia would not tell staff she was in pain and would not allow a doctor to examine her. So, potentially, she was being left with untreated pain which could have been impacting upon her behaviour.	Observation of Celia (case study 4)

Component 3: Mental health problems

Phase 1	‘The mental health problems I have seen during my time on the ward have ranged from depression and anxiety right through psychosis and terrifying hallucinations.’	Senior Nurse 4
Phase 2	‘I have seen people with visual hallucinations who have been misidentifying people and have psychotic symptoms. It all adds to the complexity’.	Psychologist
Phase 3	‘Brian’s (case study 1) mood fluctuates on a daily basis and it’s very, very difficult to console him when he’s very tearful. You can sit with him, but he just cries and cries and cries and I think you just have to let him get that out of his system.’	Interview with care staff

Component 4: Diet and fluid changes

Phase 2	‘Things that I feel, to me, are really important are people with dementia being able to eat, knowing when and what to eat, and when they can’t eat or recognise food.’	Staff Nurse 1
Phase 2	‘She has got a very, very sweet tooth, again very much a frontal temporal kind of presentation. So, you can get away with giving her any sort of pudding as long as it’s got massive amounts of sugar in it. Chocolate, crisps, anything like that she’s more than happy to eat.’	Senior Nursing Practitioner
Phase 3	Diet and fluid as a component of complexity was reflected in Celia, case study 3. During my observations of Celia, I witnessed staff, on numerous occasions, trying to get her to accept diet and fluids from them with little or no success. The most I observed her accepting from the ward staff was a couple of chips, yet she would accept food and drinks from her close family. The following extract from the data reflects the concern that her Named Nurse Linda experienced in trying to get Celia to accept food and fluids:<br><br>‘It’s the eating and drinking that really bothers me; not to have anything to eat or drink off us. It’s so fixed that, and no matter which way you go it’s so hard to… well, we’ve just not been able to break it down at all or get past it or find out why… why she’s so determined not to.’	Observation and interview with named nurse, Linda (case study 3)

Component 5: Impaired self-care ability

Phase 2	‘The majority of our patients need full assistance. Some just need prompts, but I’d say a good 80 per cent of the patients on the ward need full assistance with diet and fluids, washing and dressing, bathing.’	Staff Nurse 3
Phase 2	‘She now misidentifies anybody’s interaction with her physically and despite being in her late 70s, is actually relatively physically fit and will not, to this day, allow people to actually support her personal hygiene. Consequently, numerous members of staff have been injured. It can take three to five staff just to get her up and dressed and showered in the morning.’	Ward Manager 1
Phase 3	All four case studies in phase 3 were described by the clinical staff interviewed as requiring assistance with washing, dressing, toileting and changing continence aids. Interviews with care staff for each of the case studies identified that staff assistance in personal care was frequently refused, or resisted, and at times had to be undertaken using restraint.	Lead researcher/author notebook entry

Component 6: Sensory impairment

Phase 1	‘Visual impairment, sensory issues.’	Consultant Geriatrician 1
Phase 2	‘Now when he comes on to the dementia assessment ward you find another layer of complexity in the sense that this gentleman has profound sensory impairment, and I must confess that was the first time I had looked after someone as an inpatient like that. So the added complexity in this case was that profound sensory impairment and neither I nor the team were equipped to look after someone like that. So that adds another layer of complexity.’	Consultant Psychiatrist 1
Phase 2	‘A good example might be someone who has a visual impairment, or auditory impairment, so they’re not able to see who is approaching to initiate the therapeutic relationship. They are more likely to present with a lot of other signs and symptoms of dementia too.	Staff Nurse 2

Component 7: Mobility changes

Phase 2	'Mobility problems are a huge issue for us. You risk falls, fractured femurs. Quite often they seem to be ready for discharge and then have a fall.'	Staff Nurse 3,
Phase 2	'The main risk on the ward at the moment is the risk of falls and people with dementia being unsteady on their feet, you know. So we have to manage that by either being with them on a one-to-one basis and give them special observations or put them on observations every 10 or 15 minutes. At extremes, we have had to manage mobility changes by getting a patient to wear a safety helmet in case they fall and bang their head.'	Ward manager 2
Phase 3	During all of the observation conducted in phase 3, there was always a minimum of two people receiving enhanced observation levels (including continuous one to one observation) due to increased risk of falls as a result of mobility changes.	Lead researcher/author notebook entry

Component 8: Sleep changes

Phase 2	'We have people that don't sleep at night and then down the line you find out that they've worked nights for years, so it is important to have background information.'	Deputy Ward Manger 1
Phase 2	'Yes, there was one lady, and she just couldn't go to sleep. You would see her falling asleep and then she'd wake herself up again and all you wanted was for her to be able to rest. She was a high risk of falling because she would fall asleep standing up. We ended up following her around the ward because you didn't want her to fall. That was really difficult. We were trying all sorts of things to encourage her to be able to rest, but she just didn't want to.'	Staff Nurse 1

Component 9: Frailty

Phase 2	'Because of the risk of falls, and when older people fall and break their hip or their leg or something that's a really serious. Younger people would recover much more quickly, but for a lot of older people it can be really serious.'	Staff Nurse 1
Phase 2	'The combination of physical frailty with the	Consultant Psychiatrist 1

	presence of dementia can be defined as complex'.	
Phase 3	This added component of frailty to a dementia was reflected in case study 2 (Eric). At the time I observed Eric he was believed by the clinical team to be reaching end-of-life. His picture of complexity had evolved and he had become much weaker and frailer with increased physical health needs.	Lead researcher/author notebook entry (case study 2)

Component 10: Swallowing difficulties

Phase 2	'There was an issue around his eating because he wasn't allowed to swallow but he really liked chocolate so we tried to find some liquid chocolate as well that would make it easier.'	Psychologist

Component 11: Medication issues

Phase 2	'On the ward, I think sometimes people don't recognise that it is medication any longer. I think that maybe often it's not inviting and they don't recognise it that it is something that is going to do them some good. They can't remember that they used to take it and it is hard to rationalise [with the person with dementia] why they need to take this now and within a certain time limit.'	Ward Manager 2
Phase 3	'He [Eric, case study 2] was on covert medication. He didn't like taking medication and we got that from his wife. He never liked taking medication'.	Interview with Consultant Psychiatrist (case study 2)

Component 12: Environmental effects

Phase 1	'The ward environment can make things highly complex, for example the levels of noise, light, heat and the effect that this has on the person with dementia.'	Academic 3
Phase 1	'Dementia becomes worse on the ward because of the unfamiliarity of the environment, the number of strangers, the noise, the unexpected, the complexity of any task in an unfamiliar place... and that is quite apart from any other illness.'	Person Living with Dementia
Phase 2	'It [the ward environment] will definitely play a part in their presentation, their reaction to their	Allied Health Professional 2

	environment. Everybody's different obviously and everybody wants a different type of environment and that can be quite difficult can't it when you've got a ward full of different individuals with different needs. Some people enjoy and respond well to music and stimulus and other people would not like that and it could make them more agitated.'	
Phase 3	Whilst all cases in the case studies had difficulty with the environment, for Brian (case study 1) his behaviour and actions in this space were reflective of a man ill at ease and who knew he was not in a familiar space. As a result, Brian constantly appeared anxious. Although the ward practitioners were able to verbalise an awareness of the impact the environment can have and contribute towards complexity, in the case of Brian, this awareness did not seem to fully translate into practice. For example, Brian's care record made no mention about the effect the environment was having on him.	Lead researcher/author notebook entry (case study 1)

Component 13: Family involvement

Phase 1	'Complex family dynamics impacting upon the problems, particularly disagreements about management and care options.'	Clinical Psychologist
Phase 2	'So the family, we've had several meetings with the family and they are part of the complexity, basically. As a ward team we keep saying to them 'This is where your mother needs to go' and they have gone to every single place that's not where she's supposed to go. They're going and looking in all the residential homes where we're saying 'It's dementia nursing or a specialist behaviour unit.'	Consultant Psychiatrist 2

Component 14: Attitude and approach of others

Phase 1	'Lack of awareness and understanding on the part of the carer about how the person is being impacted cognitively.'	Academic 4
Phase 2	'Sometimes relatives are not great with their relative, and they can say things that upset them, like, remind them 'Oh no, it wasn't that mum, no, no, it's Tuesday not Saturday', you know, maybe not understanding dementia. And you can see them all becoming upset, and then when they	Staff Nurse 1

	leave the relative is walking round distraught and upset. That's difficult because you want them to have visitors, you want people to see their family, but it's not always maybe the best thing for their mental wellbeing or their mood at that time. Yes, for me, family is a big one.	
Phase 2	'I think sometimes how a member of staff approaches a person with dementia on the ward is important. Its best not to walk up to someone with dementia quickly and start talking – that can be quite frightening, I think. A member of staff's interaction with a person can start off something unwanted unless we are careful.'	Nursing Assistant 3

Fluctuating domain of complexity

Phase 1	'Complexity can go up or down, absolutely'	Academic 5
Phase 2	'In my experience, complexity changes. So, if you can remedy one of the things that's wrong then that leaves you with less things to address and it starts to become less complex. But it changes, there might be another thing added in, someone might get an infection, or something else happens, and it all changes again.'	Allied Health Professional 3
Phase 3	In the case of Charlotte (case study 3), when I interviewed her named nurse, Linda, she informed me that with regard to complexity, her changed behaviour (Fixed component 5) had appeared to reduce in acuity since she had been admitted, as she discussed:<br><br>'She's certainly a lot more settled. The periods of shouting and agitation are few and far between now really.'	Lead researcher/author notebook entry (case study 3)
